# Neuroinflammatory CSF biomarkers MIF, sTREM1, and sTREM2 show dynamic expression profiles in Alzheimer’s disease

**DOI:** 10.1186/s12974-023-02796-9

**Published:** 2023-05-05

**Authors:** Yanaika S. Hok-A-Hin, Marta del Campo, Walter A. Boiten, Erik Stoops, Melanie Vanhooren, Afina W. Lemstra, Wiesje M. van der Flier, Charlotte E. Teunissen

**Affiliations:** 1 Neurochemistry Laboratory, Department of Laboratory Medicine, Amsterdam Neuroscience, VU University Medical Center, Amsterdam UMC, Amsterdam, The Netherlands; 2 Departamento de Ciencias Farmacéuticas y de la Salud, Facultad de Farmacia, Universidad San Pablo-CEU, CEU Universities, Madrid, Spain; 3 Barcelonaβeta Brain Research Center (BBRC), Pasqual Maragall Foundation, Barcelona, Spain; 4 ADx NeuroSciences, Ghent, Belgium; 5 Department of Neurology, Alzheimer Center Amsterdam, Amsterdam Neuroscience, Vrije Universiteit Amsterdam, Amsterdam UMC, Amsterdam, The Netherlands; 6 Department of Epidemiology and Data Science, VU University Medical Centers, Amsterdam, The Netherlands

**Keywords:** Alzheimer’s disease, Neuroinflammation, CSF

## Abstract

**Background:**

There is a need for novel fluid biomarkers tracking neuroinflammatory responses in Alzheimer’s disease (AD). Our recent cerebrospinal fluid (CSF) proteomics study revealed that migration inhibitory factor (MIF) and soluble triggering receptor expressed on myeloid cells 1 (sTREM1) increased along the AD continuum. We aimed to assess the potential use of these proteins, in addition to sTREM2, as CSF biomarkers to monitor inflammatory processes in AD.

**Methods:**

We included cognitively unimpaired controls (*n* = 67, 63 ± 9 years, 24% females, all amyloid negative), patients with mild cognitive impairment (MCI; *n* = 92, 65 ± 7 years, 47% females, 65% amyloid positive), AD (*n* = 38, 67 ± 6 years, 8% females, all amyloid positive), and DLB (*n* = 50, 67 ± 6 years, 5% females, 54% amyloid positive). MIF, sTREM1, and sTREM2 levels were measured by validated immunoassays. Differences in protein levels between groups were tested with analysis of covariance (corrected for age and sex). Spearman correlation analysis was performed to evaluate the association between these neuroinflammatory markers with AD-CSF biomarkers (Aβ42, tTau, pTau) and mini-mental state examination (MMSE) scores.

**Results:**

MIF levels were increased in MCI (*p* < 0.01), AD (*p* < 0.05), and DLB (*p* > 0.05) compared to controls. Levels of sTREM1 were specifically increased in AD compared to controls (*p* < 0.01), MCI (*p* < 0.05), and DLB patients (*p* > 0.05), while sTREM2 levels were increased specifically in MCI compared to all other groups (all *p* < 0.001). Neuroinflammatory proteins were highly correlated with CSF pTau levels (MIF: all groups; sTREM1: MCI, AD and DLB; sTREM2: controls, MCI and DLB). Correlations with MMSE scores were observed in specific clinical groups (MIF in controls, sTREM1 in AD, and sTREM2 in DLB).

**Conclusion:**

Inflammatory-related proteins show diverse expression profiles along different AD stages, with increased protein levels in the MCI stage (MIF and sTREM2) and AD stage (MIF and sTREM1). The associations of these inflammatory markers primarily with CSF pTau levels indicate an intertwined relationship between tau pathology and inflammation. These neuroinflammatory markers might be useful in clinical trials to capture dynamics in inflammatory responses or monitor drug–target engagement of inflammatory modulators.

**Supplementary Information:**

The online version contains supplementary material available at 10.1186/s12974-023-02796-9.

## Introduction

Alzheimer’s disease (AD) is the most common form of dementia accounting for 70% of demented people. Dementia affects more than 50 million people worldwide with numbers rising every year [1]. Another common form of dementia in elderly is dementia with Lewy bodies (DLB), which can have AD co-pathology and overlapping clinical features [2]. The measurement of amyloid-beta (Aβ), total tau (tTau), and phosphorylated tau at threonine 181 (pTau) in the cerebrospinal fluid (CSF) are now implemented in many clinics to support AD diagnosis [3]. These biomarkers reflect the presence of Aβ plaques, neurofibrillary tangles, and neurodegeneration, which are represented in the A/T/N research framework [4]. However, other biological processes such as inflammation are also involved in AD pathology [4, 5]

Neuropathological and genetic studies have shown that immune dysfunction and inflammation are involved in the etiology of AD [6–9]. For example, variants in triggering receptor expressed on myeloid cells 2 (*TREM2*) gene increases the risk of AD [8, 9]. The soluble form of TREM2 (sTREM2) was shown to be increased in the CSF of mild cognitive impairment (MCI) and AD patients compared to controls, probably reflecting a TREM2-dependent microglia response [10–14]. Additional fluid biomarkers capturing the dynamics of disease-associated microglia and its specificity toward AD are needed to understand the disease etiology or monitor the effects of treatments [4, 15–17].

To identify AD-specific protein signatures, we used a high-throughput proteomics platform to measure > 600 proteins in CSF of a well-characterized dementia cohort including controls, MCI, AD, and DLB patients [18]. We observed that inflammatory proteins such as macrophage migration inhibitory factor (MIF) and soluble triggering receptor expressed on myeloid cells 1 (sTREM1) were among the strongest dysregulated proteins in AD (i.e., top 35 out of 288 dysregulated proteins compared to controls). Furthermore, MIF and sTREM1 were also significantly changed between patients with AD and DLB [18].

MIF is a pro-inflammatory cytokine expressed in different tissues and shown to promote the production of many other immune mediators such as cytokines [19]. Previous studies corroborate the increased CSF MIF levels in AD and MCI patients compared to controls [20–23]. Furthermore, animal models show that tau phosphorylation was attenuated in MIF-deficient mice, suggesting that MIF has potential as a therapeutic target in AD [21, 24].

TREM1 is a receptor mainly expressed by monocytes and microglial cells [25]. Levels of sTREM1 were increased in plasma of AD patients compared to controls [26], but to our knowledge, no studies reported yet on levels in CSF. Variants in the *TREM1* gene have been associated with neuritic and amyloid plaque formation and an increased rate of cognitive decline [27]. TREM1 and TREM2 belong to the same protein family and they have some similarities regarding their signaling processes. However, their function and expression profiles may differ depending on the ligand which activates them [28]. In a model for acute brain inflammation induced by lipopolysaccharide injection in mice, *TREM1* expression in microglial cells was increased, while *TREM2* expression was suppressed, and the data together suggested that *TREM1* acts as a positive regulator and *TREM2* as a negative regulator of the inflammatory response, in line with results of several other reports [28, 29].

Considering the potentially different roles of these proteins within inflammatory processes and the protein changes observed in our proteomic discovery study, we hypothesized that these inflammatory proteins may show different expression profiles in CSF across different AD stages (i.e., controls, MCI and AD). Here, we aimed to determine the potential use of MIF, sTREM1, and sTREM2 as CSF biomarkers to monitor inflammatory processes specific for AD. We tested the three inflammatory proteins in CSF from patients across different AD stages and compared the trajectories to non-AD dementia (i.e., DLB) to assess its specificity for clinical AD. Furthermore, we determined their associations with the AD pathological hallmarks and measurements for cognitive impairment in the total cohort and across clinical groups.

## Materials and methods

### Human CSF samples

Individuals with MCI (*n* = 92), AD (*n* = 38), DLB (*n* = 50), and cognitively unimpaired controls (*n* = 67) were selected from the Amsterdam Dementia Cohort [30, 31]. From this, a subset of cases was also included in our previous discovery study (*n* = 79) [18]. Individuals underwent cognitive and neurological assessments and diagnoses were made in a multidisciplinary consensus meeting according to applicable criteria [32–34]. All patients included in this study, fulfilled diagnostic criteria of probable AD [34] or probable DLB [33]. Levels of core AD-CSF biomarkers; Aβ42, tTau, and pTau were analyzed using commercially available ELISA kits [Innotest Aβ(1–42), Innotest hTAUAg, Innotest phospho-Tau(181P), Fujirebio, Ghent, Belgium]. Individuals with AD dementia were selected based on positive AD-CSF biomarker profiles as determined by increased tTau/Aβ_42_ ratio using pre-defined cut-off values (> 0.52) [35]. The control group consisted of individuals with subjective cognitive decline and was defined when clinical, cognitive testing, and biochemical assessments were within normal limits. In addition, these cases did not meet the criteria for MCI, dementia, or any other condition causing cognitive decline [36]. Furthermore, controls were selected based on a negative AD-CSF biomarker profile (tTau/Aβ_42_ ratio < 0.52) [35]. Stratification based on amyloid status or A/T classification was determined by CSF Aβ42 (positive < 813 pg/mL and negative > 813 pg/mL) and CSF pTau (positive > 52 pg/mL and negative < 52 pg/mL) [4, 37]. CSF measurements of tTau reflecting neuronal injury (“N”) was excluded from A/T stratified analysis considering the high correlation with CSF pTau measurements.

CSF was obtained by lumbar puncture from the Intervertebral space L3–L5 and collected in polypropylene tubes (Sarstedt, Germany). CSF was centrifuged 2000 × *g* for 10 min at room temperature and aliquoted in polypropylene tubes. CSF aliquots were stored at − 80 °C until biomarkers analysis following consensus guidelines [38, 39]. Informed consent was obtained from all participants or their authorized representatives, in accordance with the ethical consent by the VU University Amsterdam and with the Helsinki Declaration of 1975.

### CSF biomarker analysis

MIF and sTREM1 concentrations in CSF were determined with commercial assays (MIF: SPCKB-PS-000512 and sTREM1: SPCKB-PS-001020) on the Ella™ instrument (ProteinSimple, CA, USA) according to the manufacturer’s instructions. First, the assays were analytically validated in-house for measurements in CSF by testing the parallelism, dilution linearity, recovery, and intra- and inter-assay variation, following international guidelines for immunoassay validation [40]. A detailed overview of the analytical validation for these assays is presented in Additional file 1: Table S1 and Fig. S1. For the analysis of clinical samples, CSF was diluted two-fold in sample diluent buffer (SD13, ProteinSimple). MIF and sTREM1 assays were performed in parallel from the same samples and analyzed in triplicate.

MIF and sTREM1 protein levels in CSF measured by these immunoassays were compared to previous proteomics findings using Spearman *rho* correlation analysis in a subset of patients (18 controls, 21 MCI, 17 AD, and 23 DLB patients) [18].

The measurement of sTREM2 in CSF was determined by a prototype sandwich colorimetric ELISA, developed by ADx NeuroSciences and performance according to their protocol (Ghent, Belgium). Analytical validation is described in Additional file 1: Table S1, Fig. S1. For the analysis of clinical samples, CSF was diluted four-fold in sample diluent (ADx) and measured in duplicate.

### Statistical analysis

Statistical analysis and drafting of the figures were carried out using R Studio version 4.0.3. The normality of the data was assessed using a Shapiro–Wilk test. The influence of potential covariates such as age and sex on the biomarker levels were tested by linear regression analysis. Differences in biomarker levels between clinical groups were evaluated by ANCOVA adjusted for age or sex, when applicable, using log-transformed values. This was followed by post-hoc pairwise comparison, which was corrected for family wise error rate using the Bonferroni method. In addition, comparison of amyloid or A/T status within clinical groups was determined by linear regression or ANCOVA analysis. Associations between raw values of inflammatory proteins and CSF biomarkers or MMSE scores were assessed by Spearman *Rho* correlation analysis.

## Results

### Cohort characteristics

Demographics and clinical characteristics are listed in Table 1. Sex distribution varied between clinical groups, with fewer female patients in the AD and DLB groups compared to the control and MCI groups. The AD and DLB patients were older than controls. MMSE scores were highest in the controls compared to other groups. CSF Aβ42 was lowest, and CSF tTau and pTau were highest in the AD group. A positive amyloid status was observed in 65% of MCI and 54% of the DLB cases.

**Table 1:** Cohort characteristics

	Controls	MCI	AD	DLB
*N*	67	92	38	50
Females (%)	16 (24%)	43 (47%)	3 (8%)	5 (10%)
Age	63 (58–70)^b,c^	67 (60–71)	68 (64–72)^a^	67 (63–74)^a^
MMSE	28 (27–30)^a,b,c^	27 (26–28)^b,c^	22 (18–26)^a,d^	23 (21–26)^a,d^
Positive amyloid status (%)	0 (0%)	59 (65%)	38 (100%)	27 (54%)
CSF Aβ42, pg/mL	1088 (994–1234)^a,b,c^	730 (638–974)^b, d^	620 (577–661)^a,c,d^	790 (631–1033)^b,d^
CSF tTau, pg/mL	211 (161–263)^a,b,c^	445 (267–660)^b,c,d^	598 (495–786)^a,c,d^	305 (230–371)^a,b,d^
CSF pTau, pg/mL	44 (37–51)^a,b^	69 (46–86)^b,c,d^	79 (65–97)^a,c,d^	48 (34–64)^a,b^

Continuous data is represented as median ± interquartile range and dichotomous data as the number of cases with a percentage of the total (%)

Positive amyloid status was determined by CSF Aβ42 profile < 813 pg/mL

Differences between groups were determined using the Kruskal–Wallis test with Bonferroni correction or Chi-squared test

MCI, mild cognitive impairment; AD, Alzheimer’s disease; DLB, dementia with Lewy bodies; MMSE, mini-mental state examination; Aβ42, amyloid-beta 1–42; tTau, total tau; pTau, phosphorylated tau

^a^*p* < 0.05 compared to MCI

^b^*p* < 0.05 compared to AD

^c^*p* < 0.05 compared to DLB

^d^*p* < 0.05 compared to controls

### Validation of the immunoassay finding with the previous proteomics results

For MIF and sTREM1, the pattern of increases in the clinical groups (see below) was similar to our proteomic discovery findings [18]. In addition, strong–moderate correlations between the immunoassay and proteomics findings were observed for both proteins (MIF: *rho* = 0.622 and sTREM1: *rho* = 0.822, both *p* < 0.001, Additional file 1: Fig. S2).

### MIF, sTREM1, and sTREM2 show distinct expression profiles across AD stages and in non-AD dementia

**Fig. 1:**
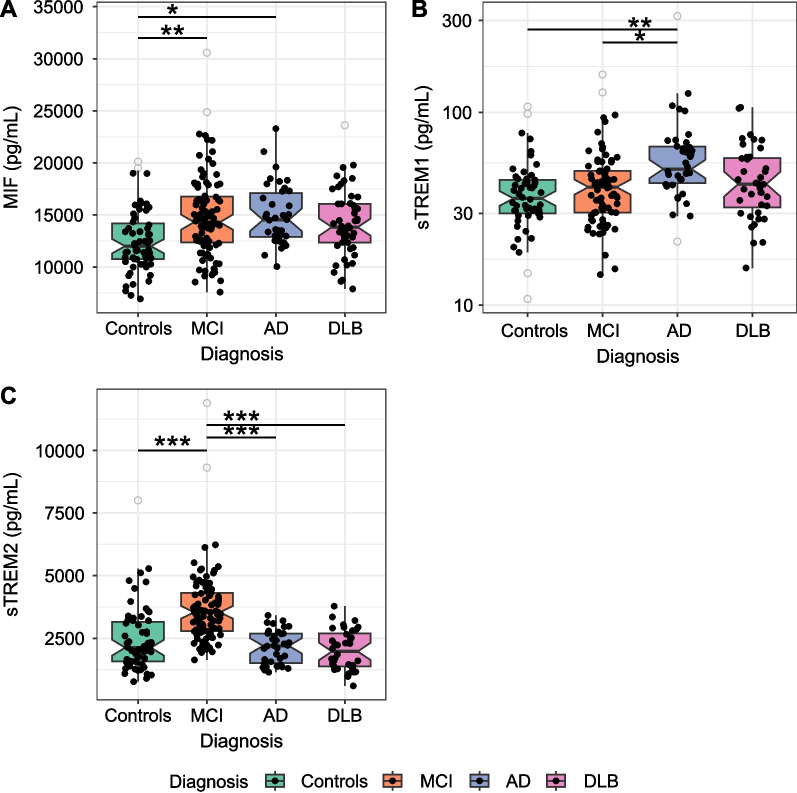
MIF, sTREM1, and sTREM2 levels in CSF are changed in different diagnostics groups. Raw values are presented and differences between groups were tested by ANCOVA adjusted for age or sex when applicable. We detected increased MIF levels in MCI and AD (**A**), sTREM1 was detected to be increased in AD (**B**), and increased sTREM2 levels were detected in MCI (**C**). Boxplots represent the median ± interquartile range. * P < 0.05, ** *P* < 0.01, *** *P* < 0.001. *MCI* mild cognitive impairment, *AD* Alzheimer’s disease, *DLB* dementia with Lewy bodies, *MIF* macrophage migration inhibitory factor, *sTREM1* soluble triggering receptor expressed on myeloid cells 1, *sTREM2* soluble triggering receptor expressed on myeloid cells 2

First, we determined the levels of inflammatory proteins in different AD stages and a non-AD dementia group to assess its specificity for AD pathology. CSF MIF levels were significantly increased in both MCI (*p* < 0.01) and AD patients (*p* < 0.05) when compared to controls, but these levels did not differ between AD and DLB patients, Fig. 1A). Increased CSF sTREM1 levels were observed in AD patients when compared to controls (*p* < 0.01), MCI (*p* < 0.05), or DLB patients (tendency: *p* = 0.07**,** Fig. 1B). CSF sTREM2 levels were specifically increased in MCI when compared to AD (*p* < 0.001), DLB patients (*p* < 0.001), or controls (*p* < 0.001, Fig. 1C). Similar trends were observed upon stratifying the MCI and DLB group for amyloid status (Additional file 1: Fig. S3). Stratifying based on A/T status showed that CSF MIF levels were increased in T + compared to T – cases in controls, MCI, and DLB groups. CSF sTREM2 levels were only increased in T + compared to T – controls. No significant trends for A/T status in clinical groups were observed for CSF sTREM1 levels (Additional file 1: Fig. S4). Overall, these inflammatory markers each showed a distinct expression profile for these different dementias.

### Association of inflammatory markers with AD-CSF biomarkers

To understand the relationship between inflammatory markers and AD pathological hallmarks, we next tested their correlation with the classical AD-CSF biomarkers.

**Fig. 2:**
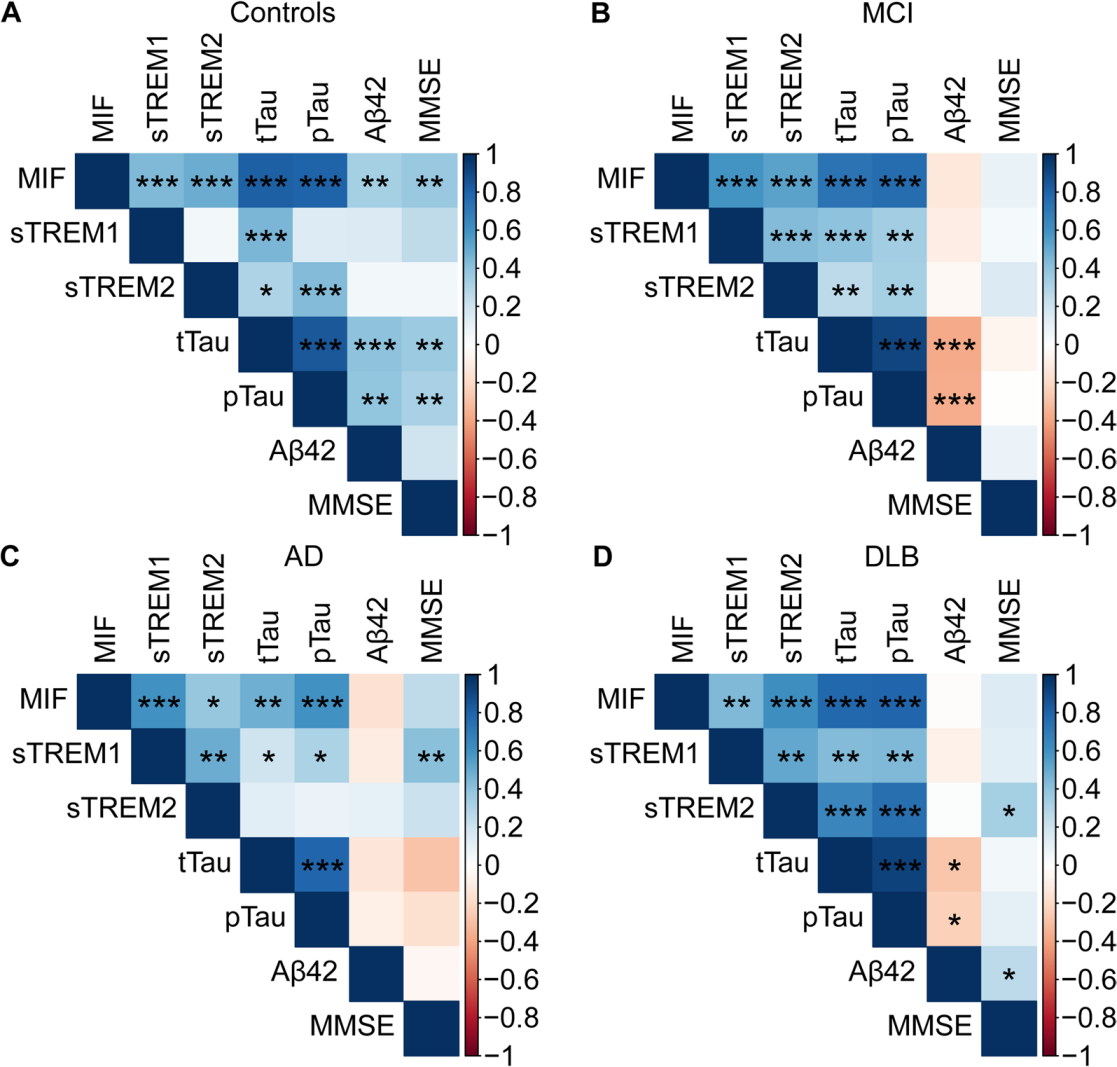
Correlation matrix showing the associations between CSF proteins in control (**A**), MCI (**B**), AD (**C**), and DLB (**D**) groups. The correlation matrix heatmap represents Spearman’s correlation coefficient between inflammatory-related proteins (i.e., MIF, sTREM1, and sTREM2), the classical AD CSF biomarkers, and MMSE scores, stratified by clinical diagnosis. The blue color depicts a positive correlation coefficient, while red depicts a negative correlation coefficient. * P < 0.05, ** *P* < 0.01, *** *P* < 0.001. *MCI* mild cognitive impairment, *AD* Alzheimer’s disease, *DLB* dementia with Lewy bodies, *MIF* macrophage migration inhibitory factor, *sTREM1* soluble triggering receptor expressed on myeloid cells 1, *sTREM2* soluble triggering receptor expressed on myeloid cells 2, *tTau* total tau, *pTau* phosphorylated tau, *Aβ42* amyloid-beta 1–42, *MMSE* mini-mental state examination

In the total cohort, MIF levels significantly correlated with AD-CSF biomarkers (Additional file 1: Fig. S5). After stratification for clinical diagnosis, moderate-to-strong correlations were detected in all groups between CSF MIF levels and tTau or pTau (Fig. 2, Additional file 1: Fig. S6). MIF levels moderately correlated with CSF Aβ42 levels in the control group (Fig. 2A), while this correlation was not observed in the other clinical groups (Fig. 2B–D).

In the total cohort, sTREM1 levels significantly correlated with all AD-CSF biomarkers (Additional file 1: Fig. S5). Upon stratification, sTREM1 levels were positively associated with tTau in all groups (Fig. 2). A positive correlation with pTau was observed in all dementia groups but not in controls (Fig. 2). Furthermore, sTREM1 levels were not associated with Aβ42 in any of the groups (Fig. 2).

In the total cohort, sTREM2 levels significantly correlated with tTau and pTau levels (Additional file 1: Fig. S5). After stratification, we observed that sTREM2 levels correlated with tTau in all groups except AD (Fig. 2C). In addition, sTREM2 did not correlate with pTau in AD (Fig. 2C), while in the other groups, moderate–strong correlations were observed (Fig. 2). sTREM2 levels did not correlate to Aβ42 in any of the groups (Fig. 2).

### Association of inflammatory markers with MMSE scores reflecting cognitive impairment

We next investigated the relationship between inflammatory markers with cognitive impairment as measured by MMSE scores. In the total cohort, only sTREM2 levels were moderately correlated with MMSE (Additional file 1: Fig. S5). Upon stratification for clinical diagnosis, MIF levels were specifically associated with MMSE scores in controls (Fig. 2A) and not in the other groups (Fig. 2B–D, and Additional file 1: Fig. S6). Moderate correlations between sTREM1 and MMSE were observed for AD patients (Fig. 2C) but not in the other clinical groups (Fig. 2D, Additional file 1: Fig. S6). Furthermore, sTREM2 was only moderately associated with MMSE scores in DLB (Fig. 2D) and not in the other groups (Fig. 2, Additional file 1: Fig. S6).

### Inflammatory markers correlate strongly with each other

To understand the relationship between these inflammatory markers, we further assessed their associations with each other. In the total group, moderate correlations were observed between all proteins (Additional file 1: Fig. S5). Stratification for clinical diagnosis showed moderate-to-strong correlations in all groups between MIF and sTREM1 or sTREM2 (Fig. 2, Additional file 1: Fig. S6). Furthermore, sTREM1 and sTREM2 levels were correlated specifically in cognitively impaired groups (Fig. 2B–D, Additional file 1: Fig. S6B–D) but not in controls (Fig. 2A, Additional file 1: Fig. S6A).

## Discussion

In this study, we show that the inflammatory proteins MIF, sTREM1, and sTREM2 have diverse CSF expression profiles along different AD stages. MIF was increased in both MCI and AD, sTREM1 was increased in AD, while sTREM2 was specifically increased in MCI patients. Stratification for A/T status showed that MIF and sTREM2 levels were increased in pTau-positive groups. In addition, these inflammatory markers associate most strongly with CSF pTau levels as AD-specific marker for tau pathology. Furthermore, the three inflammatory markers correlated moderately to strongly with each other across different AD stages and in DLB.

Inflammatory processes have been shown to contribute to AD pathogenesis and, thus, markers reflecting different aspects of the neuroinflammatory response (e.g., microgliosis and astrogliosis), when measured in CSF, could be useful to monitor specific pathophysiological mechanisms or monitoring drug–target engagement of inflammatory modulators [13, 41]. Therefore, we sought to determine whether the inflammatory proteins measured could contribute.

Here, we show that MIF levels are increased in MCI and AD stages, which is in agreement with the previous studies including our proteomics discovery study [18, 21, 22]. Interestingly, MIF was increased in AD frontal cortex and associated with amyloid plaques, suggesting that MIF levels in the CSF could reflect protein levels from the brain [21, 23]. In agreement with others, in all clinical groups, we detected that CSF MIF correlated strongly with tTau and pTau, which suggests an association with neurodegeneration and tau pathology, respectively [20, 42]. This is supported by animal studies where the deletion of the MIF protein attenuated tau phosphorylation [20, 24]. We observed a positive association of MIF to Aβ42 and MMSE scores but only in cognitively unimpaired cases, suggesting a role for MIF in normal physiological function. MIF is expressed by various cell types (i.e., neurons and glia) and is considered an early stage cytokine shown to promote the expression of other cytokines [19, 43]. Here, we observed that MIF is increased in the MCI stage and strongly associated with sTREM1 and sTREM2 in all groups, supporting the hypothesis that MIF could be part of the neuroinflammatory response cascade already in early stages and might be useful as a disease monitoring biomarker along the AD continuum.

In line with our discovery findings, sTREM1 levels were increased specifically in AD [18]. Similar results have been observed in plasma [26]. This also highlights the independent clinical validation of novel markers (MIF and sTREM1) using a different immunoassay-based technology. Genetic variants in the *TREM1* gene are associated with brain amyloidosis, as measured by Aβ PET [44]. Furthermore, experimental studies reveal that TREM1 associates with Aβ promoting microglial phagocytosis [44]. We did not observe any specific relationship between sTREM1 and Aβ42 levels, but it should be noticed that here the soluble form of TREM1 was measured, which may behave differently than the full membrane-bound TREM1 [45]. In agreement with the literature, we observed an association between sTREM1 and tau forms, suggesting a relationship between the neuroinflammatory processes and tau pathology [26]. The differences observed with our previous discovery in relation to the associations with classical AD-CSF biomarkers within the AD group might be explained by the lower sample size included in the current study [18]. CSF sTREM1 levels were positively associated with cognitive measurements in the AD group only, which is in contrast with the negative association observed in a previous study with plasma sTREM1 [26]. These discrepancies could be explained by the different matrices analyzed where sTREM1 levels in plasma are expressed by multiple sources in the periphery also showing increased levels in patients with systemic sepsis and acute myocardial infarction [46, 47], which could influence their associations with cognition. Here, sTREM1 measurements were performed in CSF, which is more likely to be brain-specific. Understanding the relations of such inflammatory markers changes over the disease course might give more detailed insight into the pathological process and etiology. The longitudinal assessment of biomarkers in AD patients would help to better define our knowledge of sTREM1 levels across the disease course and may be considered for monitoring in trials [44].

We also investigated CSF sTREM2 levels, a well-established surrogate marker for microglial functioning, and compared its trajectories to those of the novel biomarkers analyzed in this study. We observed that sTREM2 levels were specifically increased in the MCI stage, reflecting the dynamic TREM2-dependent microglial responses, which is partly in line with the previous studies [10, 11, 48, 49]. In contrast with the literature, sTREM2 levels were not increased in AD patients and no relationship with the AD-CSF biomarkers and cognition was observed specifically in the AD group [10–13, 50]. However, our study included fewer AD patients, which could explain such discrepancies. Noteworthy, in the total cohort, strong correlations with CSF pTau and tTau but not with Aβ42 were observed, which is in agreement with the literature [10–12, 50].

The observed associations between sTREM1 and sTREM2 were specific for the dementia groups. This could be due to increased shedding of these proteins during the neurodegenerative process especially considering that TREM1 and TREM2 in normal physiological function are receptor proteins [51]. Altogether, we observed that MIF, sTREM1, and sTREM2 were increased in different AD stages, which may suggest various clinical applications. MIF was increased early on and stays highly expressed in AD. Considering the multifaceted role of MIF, it could be suggested as a biomarker to monitor neuroinflammatory activation over several disease stages. The increased sTREM1 levels in AD and the specific increase of sTREM2 in MCI suggests a relation with disease stage. However, considering the fact that we also included amyloid negative MCI cases in our study that might contain different underlying pathologies, future studies should confirm our results in larger MCI amyloid positive and negative groups. Furthermore, it could be interesting to further explore how a panel of these markers would be informative for monitoring the response of anti-inflammatory drugs [14, 52].

This study is not without limitations. Our cohort included a relatively small AD group and did not reflect a broader population (i.e., equal sex distribution). We included amyloid-positive and -negative MCI patients, and thus, it cannot be excluded that we included individuals that might be on the course of developing a different type of dementia, potentially influencing our results. However, the trends of the inflammatory markers remained similar upon stratifying the groups for amyloid positivity. Another limitation of the study was that no cohort was used from an independent memory clinic, despite the fact that we validated the proteomics data using an independent method and in different patients. Future studies should also assess these markers in larger AD groups and longitudinal samples to identify the specific trajectories of these inflammatory proteins, like was already performed for the sTREM2 biomarker [10, 53]. The strengths of our study are the use of different stages of AD as well as the inclusion of a non-AD dementia group to test specificity. Furthermore, the use of technically validated assays on the automated Ella platform (MIF and sTREM1) could be beneficial to reduce inter-laboratory variations and smooth the implementation of novel biomarkers.

## Conclusions

The inflammatory markers discussed here showed diverse CSF protein levels along different AD stages. Our findings suggest that inflammation is associated mainly with CSF biomarkers reflecting either ongoing neurodegeneration or tau pathology. This data suggests that these proteins could be used to provide insight into different stages of neuroinflammatory responses. Furthermore, considering that these inflammatory markers likely reflect different inflammatory-related processes, they might be useful in clinical trials to capture the dynamics of inflammatory responses or to monitor drug–target engagement of inflammatory modulators.

## Supplementary Information

**Additional file 1: Figure S1**. MIF, sTREM1, and sTREM2 immunoassays show good analytical performance. The MIF immunoassay showed: parallelism within the acceptable criteria, no hook effect was observed, and linearity % across the dilutions is within the acceptable range. The mean recovery % of samples with low and medium spiked concentrations were not within the acceptable range. The sTREM1 immunoassay showed: parallelism within the acceptable criteria, no hook effect was observed, and linearity % across the dilutions were within the acceptable criteria. The mean recovery % of samples with low and high spiked concentrations were not in the acceptable range. The sTREM2 immunoassay showed: parallelism within the acceptable criteria, no hook effect, linearity % across the dilutions, and mean recovery % of all samples were within the acceptable ranges.** Figure S2**. MIF and sTREM1 levels are increased in AD in the discovery study. The boxplots represent the protein abundance with the median ± interquartile range observed in our proteomics discovery study. MIF levels were increased in AD and MCI-Aβ+ compared to controls and DLB patients. CSF sTREM1 is increased in AD compared to controls and DLB patients. MIF and sTREM1 measured by proteomics were moderate-strongly associated with protein levels measured by immunoassays. * P < 0.05, ** P < 0.01, *** P < 0.001. Abbreviations: MCI-Aβ+, mild cognitive impairment with amyloid pathology; AD, Alzheimer’s disease; DLB, dementia with Lewy bodies. MIF, macrophage migration inhibitory factor; sTREM1, soluble triggering receptor expressed on myeloid cells 1; sTREM2, soluble triggering receptor expressed on myeloid cells 2; NPX, normalized protein expression.** Figure S3**. MIF, sTREM1, and sTREM2 levels show a similar trend upon stratification for amyloid status. Raw values are presented and boxplots show the median ± interquartile range. Group differences were calculated based on linear regression analysis including age or sex on log-transformed values. No changes in the inflammatory proteins were observed upon stratifying the MCI and DLB group for amyloid status. Abbreviations: MCI, mild cognitive impairment; AD, Alzheimer’s disease; DLB, dementia with Lewy bodies. MIF, macrophage migration inhibitory factor; sTREM1, soluble triggering receptor expressed on myeloid cells 1; sTREM2, soluble triggering receptor expressed on myeloid cells 2.** Figure S4**. MIF levels are increased in pTau-positive groups upon A/T status stratification. Raw values are presented and boxplots show the median ± interquartile range. Differences in A/T status were calculated by linear regression analysis or by ANCOVA adjusted for age or sex, when applicable, using Log-transformed values. MIF levels were increased in T+ cases within all clinical groups. No changes in sTREM1 levels were observed while sTREM2 levels were increased in T+ cases compared to T- cases in controls. ** P < 0.01, *** P < 0.001. Abbreviations: MCI, mild cognitive impairment; AD, Alzheimer’s disease; DLB, dementia with Lewy bodies. MIF, macrophage migration inhibitory factor; sTREM1, soluble triggering receptor expressed on myeloid cells 1; sTREM2, soluble triggering receptor expressed on myeloid cells 2.** Figure S5**. Correlation matrix showing the associations between CSF proteins in the total cohort. The correlation matrix heatmap represents Spearman’s correlation coefficient between inflammatory-related proteins, the classical AD CSF biomarkers and MMSE scores in the total cohort. The blue color depicts a positive correlation coefficient, while red depicts a negative correlation coefficient with significance or the specific correlation coefficient,* rho*. ** P < 0.01, *** P < 0.001. Abbreviations: MIF, macrophage migration inhibitory factor; sTREM1, soluble triggering receptor expressed on myeloid cells 1; sTREM2, soluble triggering receptor expressed on myeloid cells 2; tTau, total tau; pTau, phosphorylated tau; Aβ42, amyloid-beta 1-42; MMSE, mini-mental state examination.** Figure S6**. Scatterplot showing the associations of inflammatory proteins with CSF biomarkers and MMSE scores. Correlations between inflammatory-related proteins, the classical AD CSF biomarkers and MMSE scores, stratified by clinical diagnosis. Spearman correlations were performed and the *rho* numbers are depicted. Abbreviations: MCI, mild cognitive impairment; AD, Alzheimer’s disease; DLB, dementia with Lewy bodies. MIF, macrophage migration inhibitory factor; sTREM1, soluble triggering receptor expressed on myeloid cells 1; tTau, total tau; pTau, phosphorylated tau; Aβ42, amyloid-beta 1-42; MMSE, mini-mental state examination.** Table S1**. Overview of analytical validation parameters. Table S2. Log-transformed means for adjusted models.

## Data Availability

The datasets used and or analyzed in the current study are available from the corresponding author upon reasonable request.

## Abbreviations

AD: Alzheimer’s disease
Aβ42: Amyloid-beta 1–42
CSF: Cerebrospinal fluid
DLB: Dementia with Lewy bodies
MCI: Mild cognitive impairment
MIF: Macrophage migration inhibitory factor
MMSE: Mini-mental state examination
pTau: Phosphorylated tau
sTREM1: Soluble triggering receptor expressed on myeloid cells 1
sTREM2: Soluble triggering receptor expressed on myeloid cells 2
tTau: Total tau
