# Supplementary material for: Neuroinflammatory CSF biomarkers MIF, sTREM1, and sTREM2 show dynamic expression profiles in Alzheimer’s disease
Source: J Neuroinflammation. 2023 May 5;20:107. doi: 10.1186/s12974-023-02796-9 (PMC10163795; doi:10.1186/s12974-023-02796-9)
Supplement: Supplementary file 1 — Additional file 1: Figure S1. MIF, sTREM1, and sTREM2 immunoassays show good analytical performance. The MIF immunoassay showed: parallelism within the acceptable criteria, no hook effect was observed, and linearity % across the dilutions is within the acceptable range. The mean recovery % of samples with low and medium spiked concentrations were not within the acceptable range. The sTREM1 immunoassay showed: parallelism within the acceptable criteria, no hook effect was observed, and linearity % across the dilutions were within the acceptable criteria. The mean recovery % of samples with low and high spiked concentrations were not in the acceptable range. The sTREM2 immunoassay showed: parallelism within the acceptable criteria, no hook effect, linearity % across the dilutions, and mean recovery % of all samples were within the acceptable ranges. Figure S2. MIF and sTREM1 levels are increased in AD in the discovery study. The boxplots represent the protein abundance with the median ± interquartile range observed in our proteomics discovery study. MIF levels were increased in AD and MCI-Aβ+ compared to controls and DLB patients. CSF sTREM1 is increased in AD compared to controls and DLB patients. MIF and sTREM1 measured by proteomics were moderate-strongly associated with protein levels measured by immunoassays. * P < 0.05, ** P < 0.01, *** P < 0.001. Abbreviations: MCI-Aβ+, mild cognitive impairment with amyloid pathology; AD, Alzheimer’s disease; DLB, dementia with Lewy bodies. MIF, macrophage migration inhibitory factor; sTREM1, soluble triggering receptor expressed on myeloid cells 1; sTREM2, soluble triggering receptor expressed on myeloid cells 2; NPX, normalized protein expression. Figure S3. MIF, sTREM1, and sTREM2 levels show a similar trend upon stratification for amyloid status. Raw values are presented and boxplots show the median ± interquartile range. Group differences were calculated based on linear regression analysis including age or sex on log-tran [file 12974_2023_2796_MOESM1_ESM.docx]

# Additional file 1

Methods

Analytical validation immunoassays

MIF, sTREM1 and sTREM2 assays were analytically validated in-house for measurements in CSF by testing the parallelism, dilution-linearity, recovery, and intra- and inter-assay coefficient of variation (CV), following international guidelines for immunoassay validation [1].

For the MIF assay (SPCKA-PS-000512, ProteinSimple), parallelism was performed by using a 2-times serial dilution of five CSF samples. Recovery was evaluated by spiking five CSF samples with a low (100 pg/mL), medium (500 pg/mL), or high (5000 pg/mL) spike of the MIF recombinant protein (898149, ProteinSimple). Dilution linearity was performed by spiking four CSF samples with MIF recombinant protein (10.000 pg/mL) following a 2-times serial dilution. Intra-assay CV% was determined by three CSF samples at the start and the end of the plate, repeated over five plates with the same lot number. Inter-assay CV% was calculated by three CSF samples repeated over five plates with the same lot number.

For the sTREM1 assay (SPCKA-PS-001020, ProteinSimple), parallelism was performed by using a 2-times serial dilution of five CSF samples. Recovery was evaluated by spiking five CSF samples with a low (10 pg/mL), medium (100 pg/mL), or high (1000 pg/mL) spike of the sTREM1 recombinant protein (898622, ProteinSimple). Dilution linearity was performed by spiking four CSF samples with sTREM1 recombinant protein (40.000 pg/mL) following a 4-times serial dilution. Intra-assay CV% was determined by three CSF samples at the start and the end of the plate, repeated over five plates with the same lot number. Inter-assay CV% was calculated by three CSF samples repeated over five plates with the same lot number.

The measurement of sTREM2 in CSF was determined by a prototype sandwich colorimetric ELISA, developed by ADx NeuroSciences and performance according to their protocol (Ghent, Belgium). Parallelism was performed by using a 2.5-times serial dilution of four CSF samples. Recovery was evaluated by spiking five CSF samples with a low (40 pg/mL), medium (200 pg/mL), or high (1000 pg/mL) spike of the sTREM2 recombinant protein (ADx NeuroSciences). Dilution linearity was performed by spiking three CSF samples with sTREM2 recombinant protein (17 ng/mL) following a 5-times serial dilution. Intra-assay CV% was determined by three CSF samples at the start and end of the plate, repeated over five plates. Inter-assay CV% was calculated by three CSF samples repeated over seven plates.

## Results

For the MIF assay, parallelism and dilution-linearity showed good analytical performance (i.e within acceptable criteria 85-115%, Supplement Figure 1A-C). Recovery analysis only CSF with high spiked samples showed good MIF protein recovery (Supplement Figure 1D). The intra-assay CV and inter-assay CV was established as 5.2% and 6.9%, respectively.

For the sTREM1 assay, parallelism and dilution-linearity showed good assay performance (Supplementary Figure 1D-F). Recovery experiments with low and high-spiked samples did not reach acceptable sTREM1 recovery, while medium-spiked samples showed sTREM1 recovery within the acceptable criteria (Supplementary Figure 1G). The intra-assay and inter-assay CV was established as 8.7% and 9.6%, respectively.

For the sTREM2 assay, analytical validation showed that parallelism, dilution-linearity and recovery parameters were within the acceptable criteria of 85-115% (Supplement Figure 1H-K). The intra-assay and inter-assay CVs were 2.1% and 8.7%. An overview of the validation parameters for these assays is presented in Supplementary Table 1.

## Results


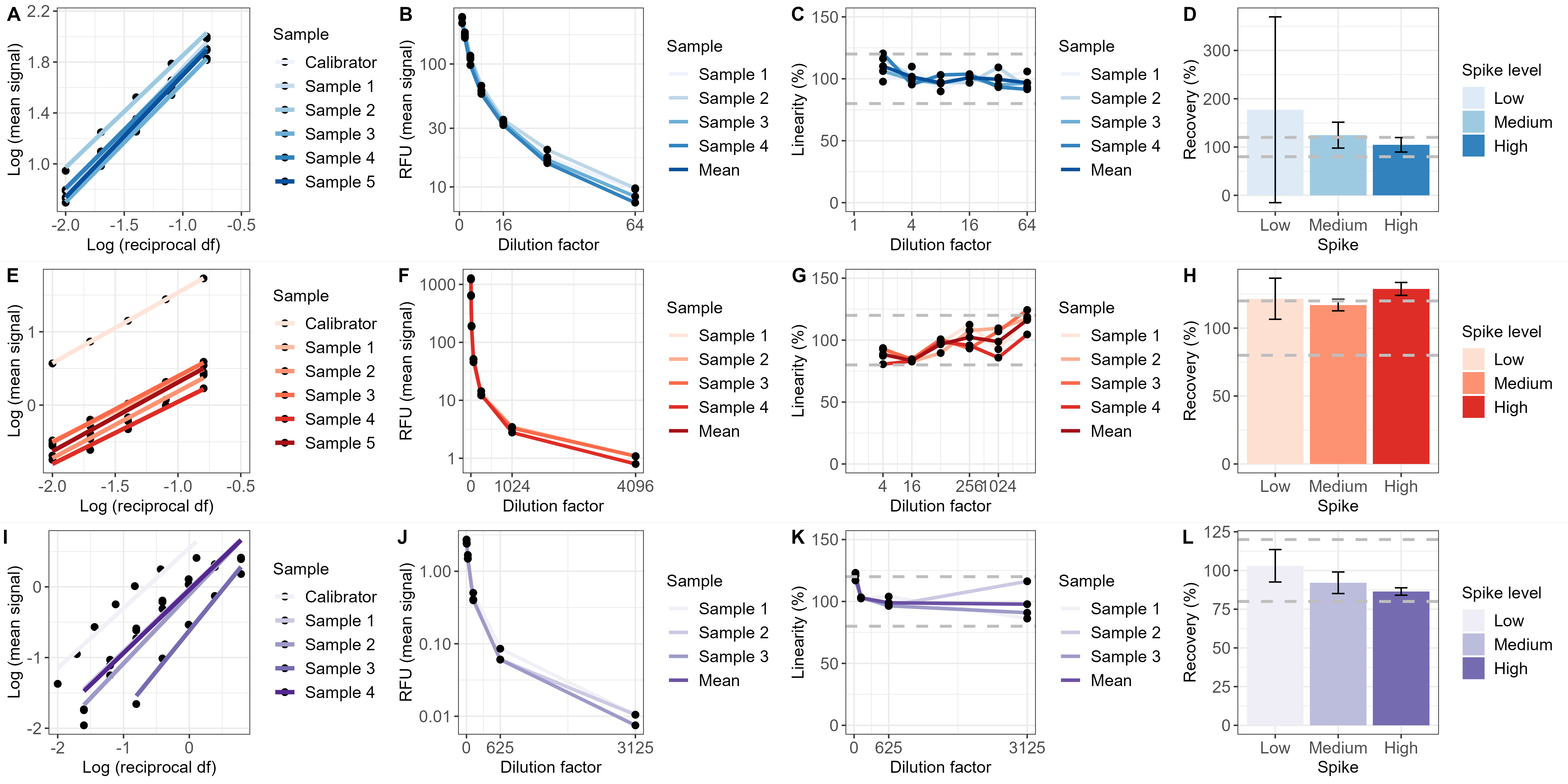


*Figure S1. MIF, sTREM1, and sTREM2 immunoassays show good analytical performance*

The MIF immunoassay (blue) showed: (A) parallelism within the acceptable criteria (85%-115%), (B) no hook effect was observed, and (C) linearity % across the dilutions is within the acceptable range. The mean recovery % (D) of samples with low and medium spiked concentrations were not within the acceptable range. The sTREM1 immunoassay (red) showed: (E) parallelism within the acceptable criteria, (F) no hook effect was observed, and (G) linearity % across the dilutions were within the acceptable criteria. The mean recovery % (H) of samples with low and high spiked concentrations were not in the acceptable range. The sTREM2 immunoassay (purple) showed: (H) parallelism within the acceptable criteria, (I) no hook effect, (J) linearity % across the dilutions, and mean recovery % (K) of all samples were within the acceptable ranges.


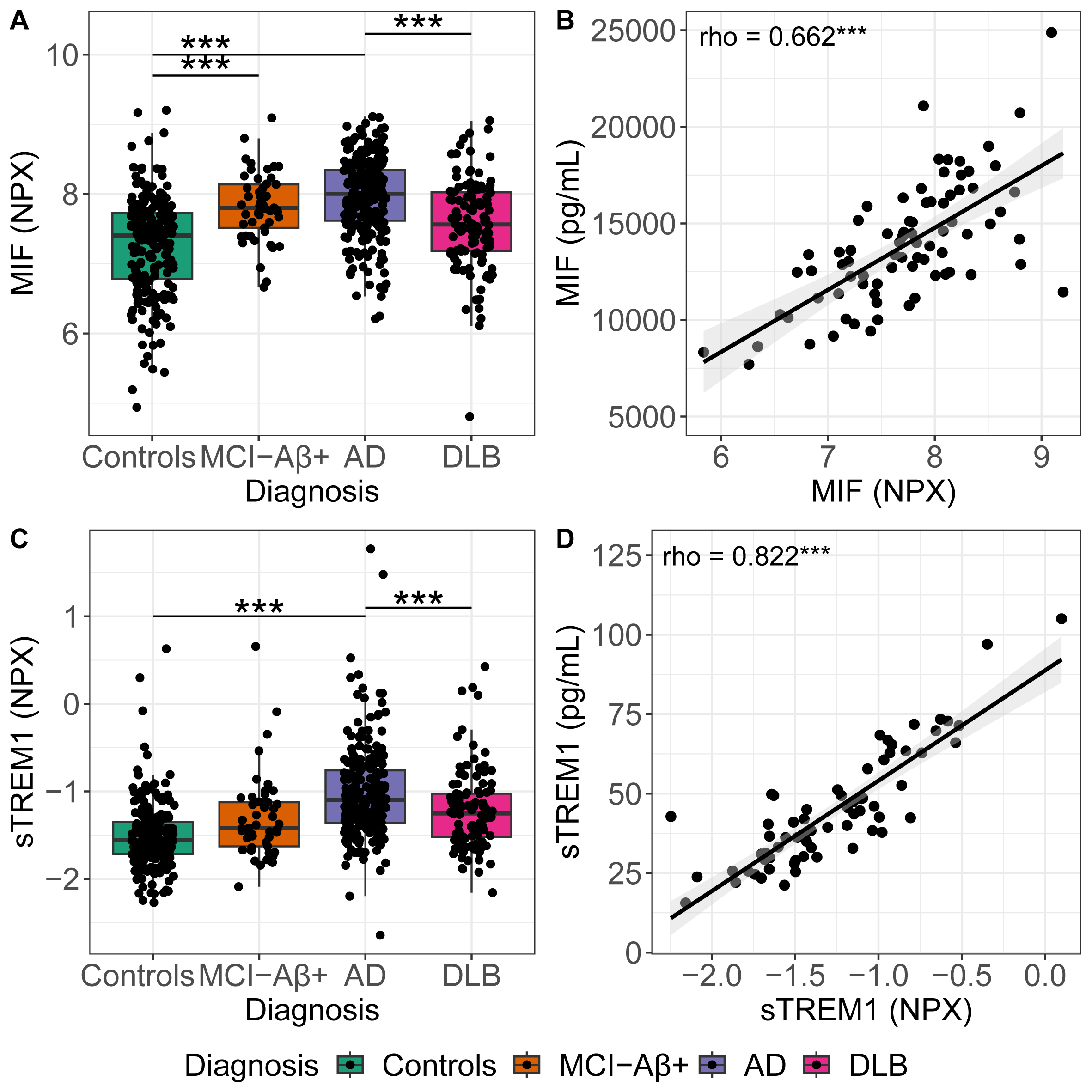


*Figure S2. MIF and sTREM1 levels are increased in AD in the discovery study*

The boxplots represent the protein abundance (log2 NPX) with the median ± interquartile range observed in our proteomics discovery study. MIF levels were increased in AD and MCI-Aβ+ compared to controls and DLB patients (A). CSF sTREM1 is increased in AD compared to controls and DLB patients (B). MIF and sTREM1 measured by proteomics were moderate-strongly associated with protein levels measured by immunoassays (B-D). * *P* < 0.05, ** *P* < 0.01, *** *P* < 0.001. Abbreviations: MCI-Aβ+, mild cognitive impairment with amyloid pathology; AD, Alzheimer’s disease; DLB, dementia with Lewy bodies. MIF, macrophage migration inhibitory factor; sTREM1, soluble triggering receptor expressed on myeloid cells 1; sTREM2, soluble triggering receptor expressed on myeloid cells 2; NPX, normalized protein expression.


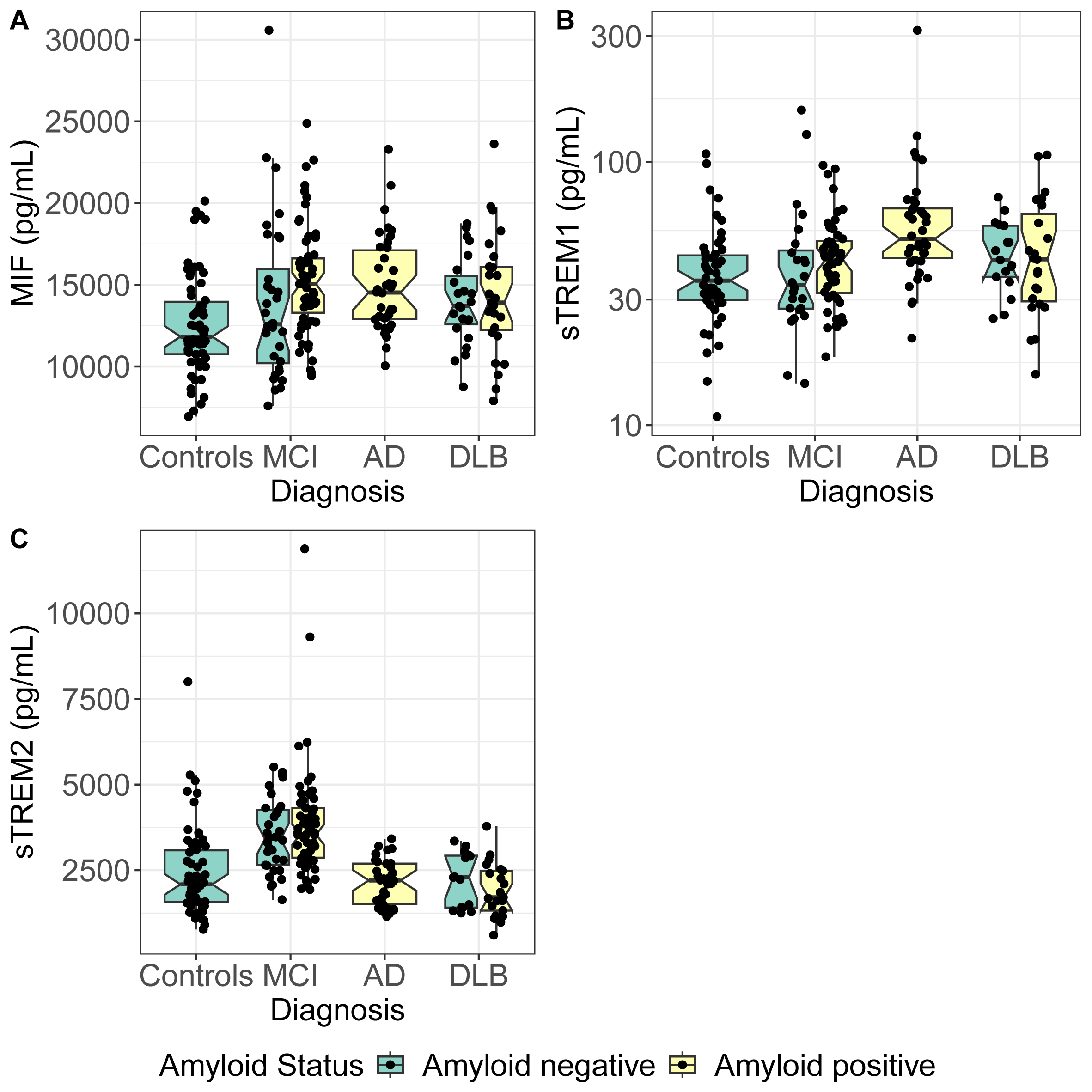


*Figure S3. MIF, sTREM1, and sTREM2 levels show a similar trend upon stratification for amyloid status.*

Raw values are presented and boxplots show the median ± interquartile range. Group differences were calculated based on linear regression analysis including age or sex on log-transformed values. No changes in the inflammatory proteins were observed upon stratifying the MCI and DLB group for amyloid status. Abbreviations: MCI, mild cognitive impairment; AD, Alzheimer’s disease; DLB, dementia with Lewy bodies. MIF, macrophage migration inhibitory factor; sTREM1, soluble triggering receptor expressed on myeloid cells 1; sTREM2, soluble triggering receptor expressed on myeloid cells 2.


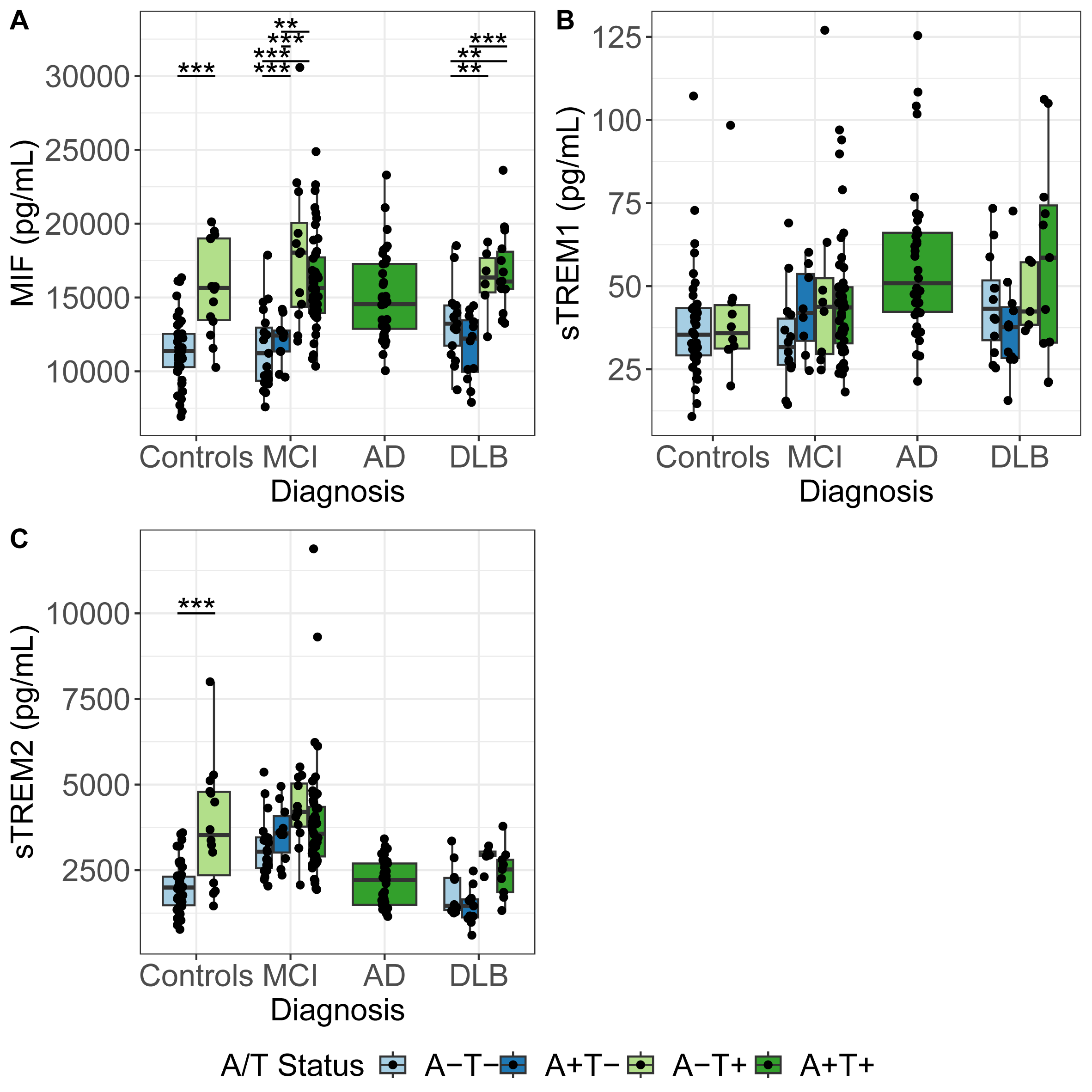


*Figure S4. MIF levels are increased in pTau-positive groups upon A/T status stratification.*

Raw values are presented and boxplots show the median ± interquartile range. Differences in A/T status were calculated by linear regression analysis (for 2 groups) or by ANCOVA (> 2 groups) adjusted for age or sex, when applicable, using Log-transformed values. MIF levels were increased in T+ cases within all clinical groups. No changes in sTREM1 levels were observed while sTREM2 levels were increased in T+ cases compared to T- cases in controls. ** *P* < 0.01, *** *P* < 0.001. Abbreviations: MCI, mild cognitive impairment; AD, Alzheimer’s disease; DLB, dementia with Lewy bodies. MIF, macrophage migration inhibitory factor; sTREM1, soluble triggering receptor expressed on myeloid cells 1; sTREM2, soluble triggering receptor expressed on myeloid cells 2


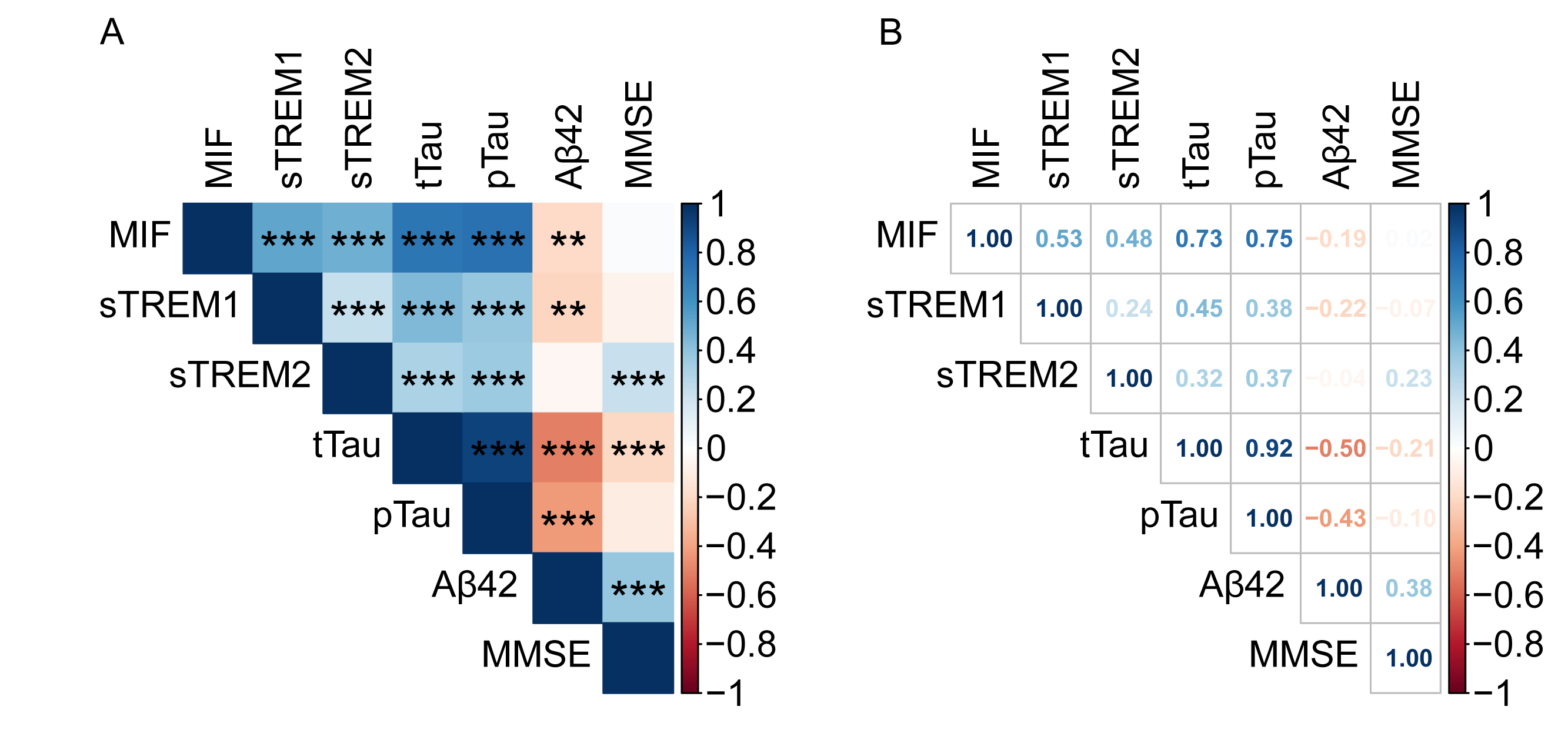


*Figure S5. Correlation matrix showing the associations between CSF proteins in the total cohort*

The correlation matrix heatmap represents Spearman’s correlation coefficient between inflammatory-related proteins (i.e., MIF, sTREM1, and sTREM2), the classical AD CSF biomarkers and MMSE scores in the total cohort. The blue color depicts a positive correlation coefficient, while red depicts a negative correlation coefficient with significance (A) or the specific correlation coefficient, *rho* (B). ** *P* < 0.01, *** *P* < 0.001. Abbreviations: MIF, macrophage migration inhibitory factor; sTREM1, soluble triggering receptor expressed on myeloid cells 1; sTREM2, soluble triggering receptor expressed on myeloid cells 2; tTau, total tau; pTau, phosphorylated tau; Aβ42, amyloid-beta 1-42; MMSE, mini-mental state examination.


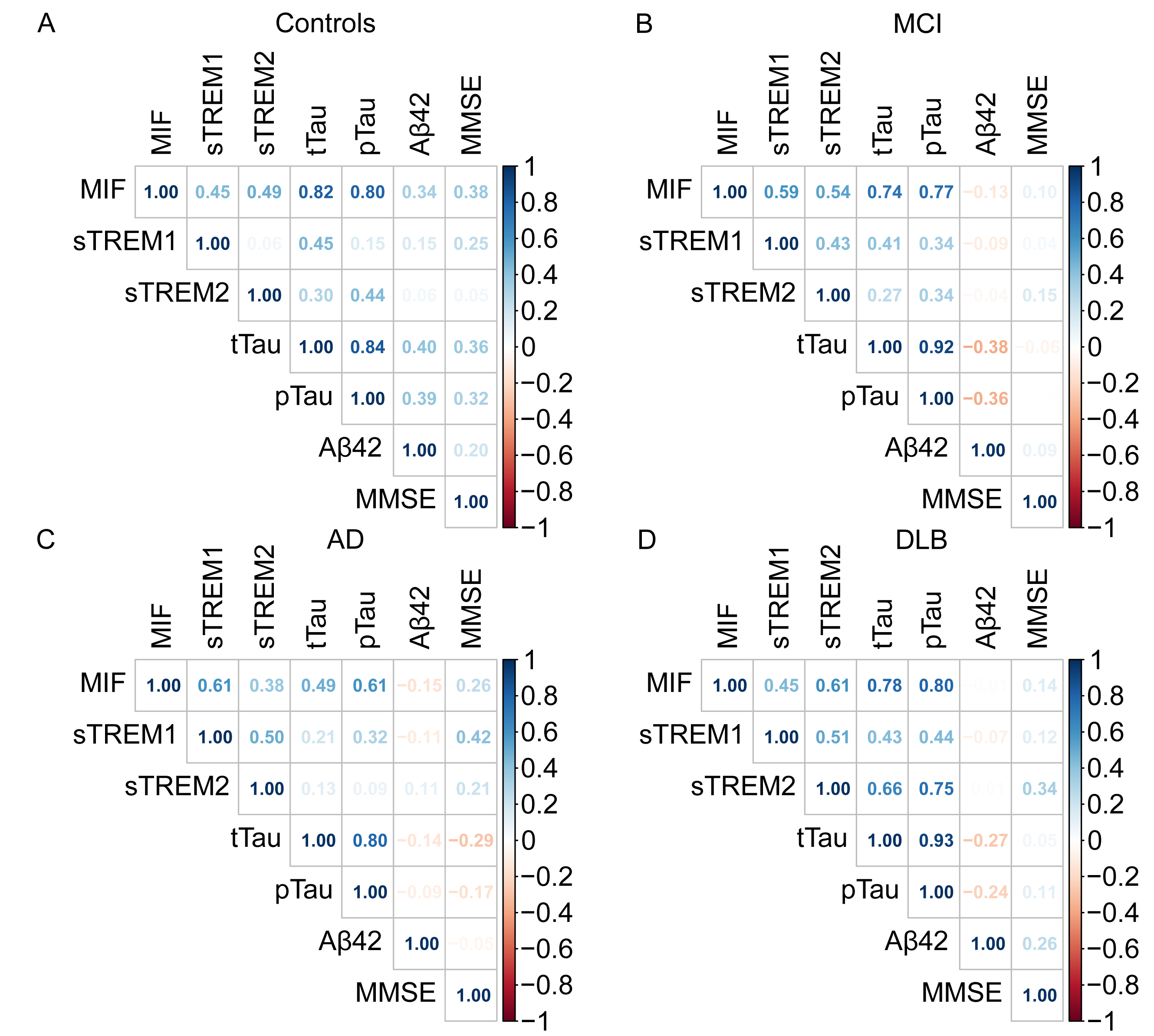
 *Figure S6. Scatterplot showing the associations of inflammatory proteins with CSF biomarkers and MMSE scores*

Correlations between inflammatory-related proteins (MIF, sTREM1, and sTREM2), the classical AD CSF biomarkers and MMSE scores, stratified by clinical diagnosis. Spearman correlations were performed and the rho numbers are depicted. Abbreviations: MCI, mild cognitive impairment; AD, Alzheimer’s disease; DLB, dementia with Lewy bodies. MIF, macrophage migration inhibitory factor; sTREM1, soluble triggering receptor expressed on myeloid cells 1; tTau, total tau; pTau, phosphorylated tau; Aβ42, amyloid-beta 1-42; MMSE, mini-mental state examination

**Table S1. Overview of analytical validation parameters**

|  | Assay: | **MIF** | | **sTREM1** | | **sTREM2** | |
| --- | --- | --- | --- | --- | --- | --- | --- |
| *Sensitivity* | LLOD | 9.3 pg/mL | | 2.2 pg/mL | | 14.0 pg/mL | |
| *Precision* | Intra-assay %CV | 5.2 | | 8.7 | | 2.1 | |
|  | Inter-assay %CV | 6.9 | | 9.6 | | 8.7 | |
| *Parallelism* | Average slope samples | 0.854 | | 0.883 | | 0.951 | |
|  | Slope calibrator | 0.881 | | 0.962 | | 0.932 | |
|  | Parallelism % | 96.9 | | 91.8 | | 102.1 | |
| *Dilution Linearity* | Spiked concentration | 10.000 pg/mL | | 40.000 pg/mL | | 173.900 pg/mL | |
|  | Dilution factor with mean % Linearity | Df (x) | Mean %L | Df (x) | Mean %L | Df (x) | Mean %L |
|  |  | 1 | - | 1 | - | 1 | - |
|  |  | 2 | 110 | 4 | 89 | 5 | - |
|  |  | 4 | 102 | 16 | 83 | 25 | 121 |
|  |  | 8 | 97 | 64 | 97 | 125 | 103 |
|  |  | 16 | 101 | 256 | 102 | 625 | 99 |
|  |  | 32 | 100 | 1024 | 99 | 3125 | 98 |
|  |  | 64 | 97 | 4096 | 116 |  |  |
| *Recovery* | Spiked concentration (pg/mL) | Spike | Mean %R | Spike | Mean %R | Spike | Mean %R |
|  | with mean % Recovery | 94 | 177 | 9 | 122 | 41 | 103 |
|  |  | 426 | 125 | 95 | 117 | 202 | 92 |
|  |  | 4749 | 105 | 9690 | 129 | 1029 | 86 |

LLOD was calculated as the mean signal from 12 blank samples plus 10 times the standard deviation with the concentrations extrapolated from the calibration curve. Intra-assay CV% was determined by taking the mean CV% of CSF samples with low, medium, and high concentrations of the analyte that were measured at the start and the end of the plate. Inter-assay CV% was determined by taking the mean CV% of CSF samples with low, medium, and high concentrations of the analyte that were measured over five independent plates. Parallelism was determined by measuring CSF samples (MIF and sTREM1 assays, N=5 and sTREM2 assay, N=4) with 2-fold serial dilution. For dilution linearity, CSF samples (MIF and sTREM1 assays, N=4 and sTREM2 assay, N=3) were spiked with a high concentration of recombinant protein and serial diluted until LLOD. For recovery, five CSF samples were spiked with low, medium, and high concentrations of recombinant protein. Abbreviations: MIF, macrophage migration inhibitory factor; sTREM1, soluble triggering receptor expressed on myeloid cells 1; sTREM2, soluble triggering receptor expressed on myeloid cells 2; %CV, % coefficient of variation; %L, % linearity; Df, dilution factor; %R, % recovery.

**Table S2. Log-transformed means for adjusted models**

|  | **Controls** | **MCI** | **AD** | **DLB** |
| --- | --- | --- | --- | --- |
| *Log MIF* | 4.10 CI: 4.07 – 4.12 | 4.16 CI: 4.14 – 4.18 | 4.16 CI: 4.12 – 4.19 | 4.32 CI: 4.14 – 4.16 |
| *Log sTREM1* | 1.59 CI: 1.54 – 1.63 | 1.61 CI: 1.57 – 1.64 | 1.70 CI: 1.65 – 1.75 | 1.61 CI: 1.56 – 1.66 |
| *Log sTREM2* | 3.35 CI: 3.31 – 3.39 | 3.53 CI: 3.50 – 3.57 | 3.30 CI: 3.25 – 3.35 | 3.28 CI: 3.22 – 3.33 |

Age or sex adjusted means from Log-transformed inflammatory markers with their respective 95% confidence interval. Abbreviations: MIF, macrophage migration inhibitory factor; sTREM1, soluble triggering receptor expressed on myeloid cells 1; sTREM2, soluble triggering receptor expressed on myeloid cells 2; CI, 95% confidence interval.

## References

1. Andreasson, U., et al., *A Practical Guide to Immunoassay Method Validation.* Front Neurol, 2015. **6**: p. 179.
